# Evaluation of urinary trace element levels in patients with opioid use disorder undergoing methadone treatment in western Iran

**DOI:** 10.1038/s41598-024-56241-9

**Published:** 2024-03-07

**Authors:** Samaneh Nakhaee, Alireza Amirabadi Zadeh, Yazdan Madadjoo, Nammam Ali Azadi, Borhan Mansouri

**Affiliations:** 1 Medical Toxicology and Drug Abuse Research Center (MTDRC), Birjand University of Medical Sciences, Birjand, Iran; 2 Endocrine Research Center, Research Institute for Endocrine Sciences, Shahid Beheshti University of Medical Sciences, Tehran, 9717113163 Iran; 3 State Welfare Organization of Kermanshah, Kermanshah, Iran; 4 Biostatistics Department, School of Public Health, Iran University of Medical Sciences, Tehran, Iran; 5 Substance Abuse Prevention Research Center, Research Institute for Health, Kermanshah University of Medical Sciences, Kermanshah, Iran

**Keywords:** Opioid use disorder, Methadone treatment, Trace elements, Heavy metals, Health care, Medical research, Risk factors, Chemistry

## Abstract

The monitoring of essential and toxic elements in patients with Opioid Use Disorder (OUD) undergoing methadone treatment (MT) is important, and there is limited previous research on the urinary levels of these elements in MT patients. Therefore, the present study aimed to analyze certain elements in the context of methadone treatment compared to a healthy group. In this study, patients with opioid use disorder undergoing MT (n = 67) were compared with a healthy group of companions (n = 62) in terms of urinary concentrations of some essential elements (selenium (Se), zinc (Zn), copper (Cu), iron (Fe), manganese (Mn), calcium (Ca)) and toxic elements (lead (Pb), cadmium (Cd), arsenic (As), and chromium (Cr)). Urine samples were prepared using the acid digestion method with a mixture of nitric acid and perchloric acid and assessed using the ICP-MS method. Our results showed that the two groups had no significant differences in terms of gender, education level, occupation, and smoking status. Urinary concentrations of Se, Cu, and Fe levels were significantly lower in the MT group compared to the healthy subjects. However, the concentrations of Pb, Cd, As, Mn, Cr, and Ca in the MT group were higher than in the healthy group (*p* < 0.05). No significant difference was established between the levels of Zn in the two groups (p = 0.232). The results of regression analysis revealed that the differences between the concentration levels of all metals (except Zn) between two groups were still remained significant after adjusting for all variables (*p* < 0.05). The data obtained in the current study showed lower urinary concentrations of some essential elements and higher levels of some toxic elements in the MT group compared to the healthy subjects. These findings should be incorporated into harm-reduction interventions.

## Introduction

Substance use disorder (SUD) is characterized by continuous seeking and using drugs despite its negative health, occupational, and social consequences^1,2^. It is a major public health issue worldwide^3^, with nearly 30 million individuals experiencing SUDs out of the approximately 250 million people who engage in the consumption of addictive substances every year^4^. In Iran, official reports indicate that around 2.1% of the population between 15 and 64 years old are currently dealing with drug use disorders, which accounts for around 1.12 million individuals, with opioid use disorder (OUD) being the predominant issue within this group affecting approximately 1.8% of the population^4,5^.

Iran shares borders with Afghanistan and Pakistan, making it a significant conduit for global drug trafficking. This proximity has facilitated convenient access to drugs and subsequently contributed to a substantial number of drug users within the country^6^. OUD can lead to a series of adverse outcomes at psychological, social, and emotional levels. It also increases the risk of some diseases and medical problems in different organs such as the liver, kidney, heart, endocrine, and nervous system^1,7^. In addition, OUD can greatly affect the dietary habits and nutritional status of individuals^7^.

Various types of substances such as opium and cannabis may cause nutrient deficiency or malnutrition, resulting in an immunodeficiency status^2^. The intake of most minerals and vitamins is below the recommended values in this population^7^. Changes in the concentration of trace elements due to substance use have been reported in previous research^2,8,9^. Trace elements such as zinc (Zn), iron (Fe), copper (Cu), and magnesium play multiple roles in the body from creating immunity to providing antioxidant defenses^2,10,11^. The lack or overload of any of these trace elements may strongly influence the usual function of the human body or cause immunotoxicity^2^.

Unfortunately, heavy toxic metal poisoning in persons with OUD has become one of the major consequences recently^12^. Vendors may add many impurities in these substances for more profit, so that the presence of high levels of lead (Pb) in methadone oral solution and opium is mentioned in the findings of Rahimi et al.^12^. Non-essential metals such as arsenic (As), lead (Pb), and cadmium (Cd) do not play a role in the normal functions of the body and may even cause harmful results on the health of exposed subjects^13–17^.

Several detoxification and rehabilitation methods are used to help people withdraw from opioid use^7^. Methadone treatment (MT) appears to be a long-acting treatment modality that replaces opioids with other safe alternatives to manage physical and psychological problems and reduce withdrawal symptoms and cravings^7,12,18–20^. Currently, some researchers have reported changes in the diet and nutritional habits of persons undergoing methadone treatment^7,21^. Essential trace element level alterations in persons who undergo MT have been documented previously with contradictory results^2,10,22^. The development of pathological conditions and diseases can occur when there are fluctuations in the levels of trace elements^2^. Some studies hypothesized that taking micronutrients can demote dependence on opioids and relapse rate, which has been supported by the low levels of this nutrient in patients with OUD^23^. Additionally, experiences of heavy metal poisoning in persons who have been treated with methadone oral solution and/or opium tincture^12,24,25^ confirm the need for research in this vulnerable group. Some limited studies have investigated toxic metal levels in Iranian persons on methadone treatment^25^.

As mentioned previously essential and toxic elements monitoring in patients with OUD undergoing methadone treatment is important and there is limited previous research on the urinary levels of these elements in MT patients. So, the goal of the present study is to analyze urinary levels of 12 essential and toxic elements in the context of MT compared with a healthy control group to contribute to research on OUD. Based on previous documents, we hypothesize that essential element levels may be decreased and some toxic elements may be increased in persons undergoing methadone treatment.

## Materials and methods

### Study design and setting

This cross-sectional study was conducted on opioid use disorder patients treated with methadone in Kermanshah city, located in the west of Iran, from April to December 2022. This study was approved by the Research and Ethics Committee of Kermanshah University of Medical Sciences (IR.KUMS.REC.1401.187). All methods were performed in accordance with the relevant guidelines and regulations. The number of OUD patients was estimated using the t-test for comparison of the mean differences of heavy metal levels between two groups (MT vs control) using the following equation:

$${n}_{1}=\frac{r+1}{r}\times \frac{{{\sigma }^{2}({Z}_{1-\beta }+{Z}_{1-\alpha /2})}^{2}}{{(d)}^{2}}$$

where ${{\text{n}}}_{1}$ is the sample size in the MT group, σ is the common standard deviation, r is the ratio of MT and control group sample sizes, and d denotes the smallest difference between the mean concentration levels of two groups considered as significant. d/σ is known as the Cohen’s d effect size of the study, α is the type I error, and 1-β is the target power of the test. Typical values of 0.05 and 0.80 were used for α and power of the test, respectively^26,27^. Assuming two equal-sized groups (r = 1) and a Cohen effect size of 0.5 (d = 0.5σ), the number of subjects estimated in each group was 62.

The selection of participants from the list of OUD patients available via methadone clinics in Kermanshah city, was conducted using a simple random sampling method. . All participants signed written informed consent.

### Study population

The study population consisted of OUD patients treated with methadone (MT group) and their companions, who were mainly one of their family members (control group). Although all patients in the MT group used opium, they were classified based on the type of drug used: (i) purely opium (22 subjects, 32.8%), (ii) opium plus tramadol (8 subjects, 11.9%), (iii) opium plus cannabis (13 subjects, 19.4%), or (iv) opium plus tramadol plus cannabis (24 subjects, 35.8%). Methadone were taken orally in the form of a liquid.The average dose of the MT was 40 mg per day for individual. The duration of methadone treatment programs ranges from a few months to years. Since controls were selected from patients' companions, we expected a good match between the two groups in terms of their lifestyle, diet, genetic characteristics, residence, and exposure to heavy metals. Individuals in the control group self-reported that they had no history of opium, tramadol, cannabis, or any other types of drug use. Exclusion criteria included: (i) current use of drugs or alcohol; (ii) history of chronic diseases; (iii) exposure to pollutants through their jobs; (iv) current use of medications; and (v) use of dietary supplements.

### Data collection

Data were collected by a trained person from registered MT centers. Participants' characteristics, including age, gender, height, weight, smoking habits, and education level, were recorded using a checklist. The type of drugs used was obtained for each subject. To evaluate the concentration levels of heavy metals for each participant, 5 ml urine samples were collected, capped, labeled, and stored in a refrigerator at −20 °C until analysis.

### Element analyses

In this study, urine samples were prepared using the acid digestion method with a nitric acid and perchloric acid mixture (2:1 v/v) to measure heavy metals and arsenic. First, the frozen samples were thawed, and 1 mL of urine sample was transferred to a test tube. Then, for mineralization, 2 mL of nitric acid (HNO_3_, 65%, Merck, Germany) was added to each sample and left in the laboratory environment for one day and night for slow acid digestion. After that, 1 mL of perchloric acid (HClO_4_, 70%, Merck, Germany) was added to each sample, and the samples were placed in a Bain-Marie (hot water bath). The samples were kept at a temperature of 95 °C for 6 h until the samples became clear. The mineralized solutions were then cooled to room temperature and made up to a volume of ten mL with ultrapure water (18.2 MΩ-cm at 25 °C, Fistreem, WSC044, UK). Before heavy metal assay by Inductively Coupled Plasma Mass Spectrometry (ICP-MS, Agilent 7900 ICP-MS), the samples were filtered. Trace elements measured included essential elements [selenium (Se), zinc (Zn), copper (Cu), iron (Fe), manganese (Mn), calcium (Ca)] and toxic elements [lead (Pb), cadmium (Cd), arsenic (As), and chromium (Cr)], with recovery rates between 97 and 103%. Spikes and control samples were run every five analyses and adjusted to fit the midpoint of the working range of the methods.

### Statistical evaluation

Descriptive statistics were obtained using either mean and standard deviations for numerical variables or frequency and proportion for nominal variables. The normality assumption was assessed using the Shapiro–Wilk test. Depending on the result of the normality test, either an independent t-test or Mann–Whitney test was used to compare summaries between the MT and control groups. Moreover, the Kruskal–Wallis test was used to compare the concentrations of heavy metals between different subgroups of drug users (four groups).Since metal concentration data are mainly right-skewed and subject to the outliers, as an alternative to the ordinary least squares regression models, a rubust regression analysis was used on the log-transformed heavey metals^28^. The correlation structure between trace elements was assessed using Spearman correlation test.

### Ethics approval and consent to participate

This study was approved by the Research and Ethics Committee of Kermanshah University of Medical Sciences (IR.KUMS.REC.1401.187).

## Results

### Participants

Table 1 reports the characteristics of study participants in both the MT and control groups. The two groups were comparable in terms of gender distribution (p = 0.75), education levels (p = 0.51), job occupation (p = 0.28), and smoking habit (p = 0.48). However, there was a significant difference between the two groups in terms of participants' age (t-test, *p* < 0.001). The mean age of participants in the MT group was 35.5 years, which was higher than the control group (29.12 years).

**Table 1:** Characteristics of participants in both methadone treatment (MT) and control groups.

Variable	MT group	Control group	P-value
Age (year)	35.53 ± 10.80	29.12 ± 7.05	< 0.001
Gender			
Male	36.0 (53.7)	36.0 (58.1)	0.751
Female	31.0 (46.3)	26.0 (41.9)	
Education			
Primary school	17.0 (25.4)	16.0 (25.8)	0.511
High school	35.0 (52.2)	27.0 (43.5)	
University	15.0 (22.4)	19.0 (30.6)	
Occupation			
Unemployed	20.0 (29.9)	19.0 (30.6)	0.281
Part-time worker	13.0 (19.4)	12.0 (19.4)	
Public sector	18.0 (26.9)	9.0 (14.5)	
Self-employed	16.0 (23.9)	22.0 (35.5)	
Cigarette smoking			
Yes	21.0 (31.3)	15.0 (24.2)	0.479
No	46.0 (68.7)	47.0 (75.8)	

The presented values are mean ± SD or number (percentage).

### Trace element concentrations

**Figure 1:**
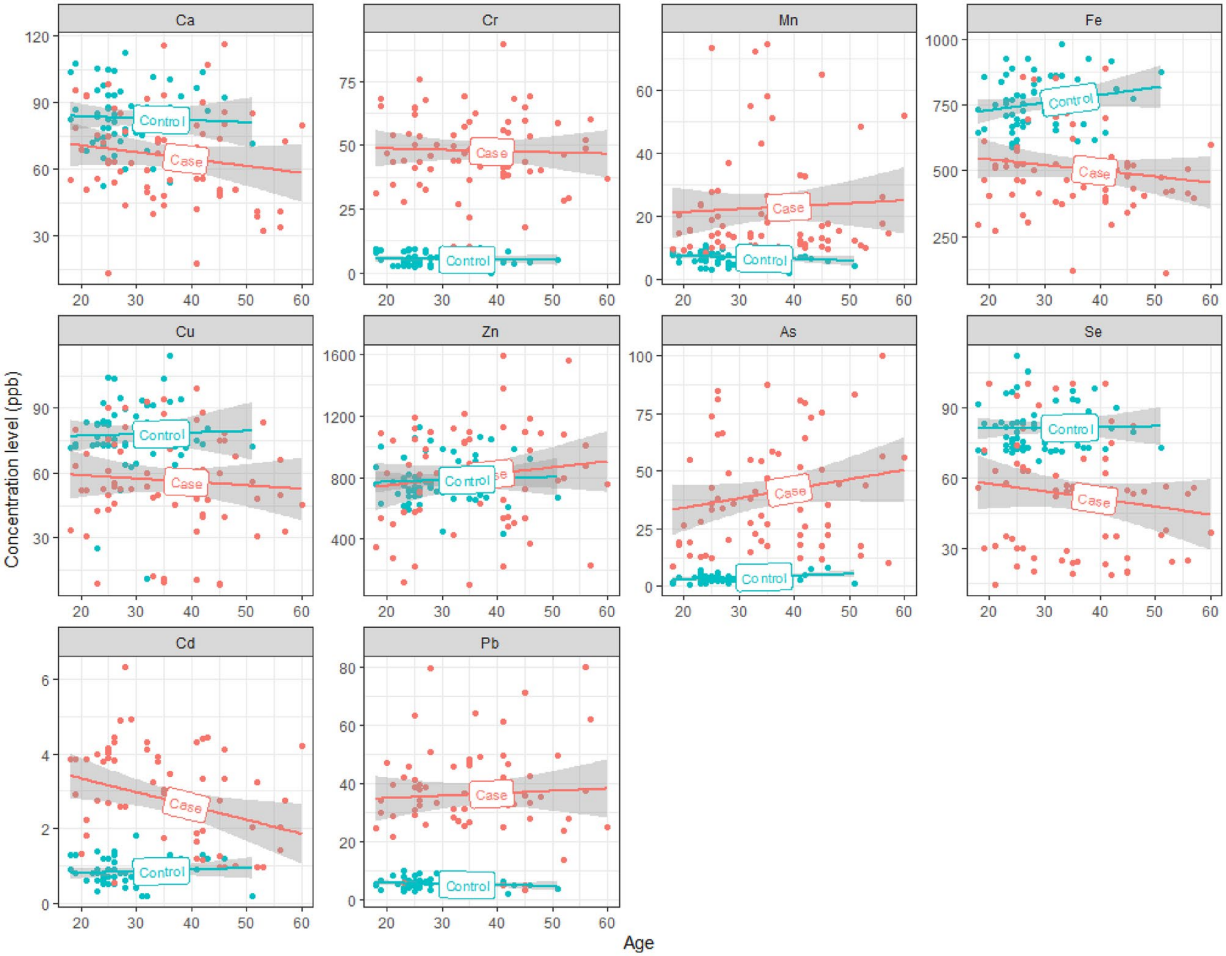
The associations between age and concentration levels of heavy metals for methadone treatment (MT) and control groups. The lines represent a linear trend for the levels of metals in a simple linear regression model with corresponding 95% confidence area.

Table 2 shows the median and quantile concentration levels of trace elements for participants in the MT and control groups. Compared to control group, OUD patients revealed markedly higher levels of non-essential heavy metals such as Pb, Cd, As, and Cr (Mann–Whitney test, *p* < 0.001). They also showed lower levels of essential elements such as Se, Cu, and Fe. However, Mn levels were higher among OUD patients compared to their controls. No significant difference in Zn levels was established between two groups (Mann–Whitney test, p = 0.232 and p = 0.081, respectively). Since two groups were different in terms of participants' age (Table 1), to investigate this further, the association between age and concentration levels of metals was investigated for both methadone treatment (MT) and control groups separately (Fig. 1). The lines in Fig. 1 represent a linear trend for the levels of metals in a simple linear regression model with corresponding 95% confidence area. As compared to the control group, the higher levels of Cr, Mn, As, Cd, and Pb as well as the lower levels of Fe, Cu, and Se can be seen among OUD patients (95% CIs are well separated). No difference in Ca and Zn levels between two groups is observed (95% CIs are overlapped). Moreover, the trends of Ca, Cr, Mn, Cu, Zn, an Pb are similar in both groups and almost constant with the age. For controls, the levels of Fe increased with age, but in OUD patients Fe levels decreased with age. The levels of As increased whereas the levels of Se and Cd declined with age in case group. Apart from the Fe levels that showed an upward trend for controls, the levels of other elements were constant with age in control group.

**Table 2:** Comparison of the heavy metal concentrations (µg L^−1^) between methadone treatment (MT) and control group.

Element	MT (n = 67)	Control (n = 62)	P-value
Lead (Pb)	35.50 [27.85–45.86]	5.10 [4.12–6.50]	** < 0.001**
Cadmium (Cd)	2.75 [1.37–3.89]	0.80 [0.60–1.10]	** < 0.001**
Selenium (Se)	55.00 [29.79–67.02]	80.80 [72.90–84.05]	** < 0.001**
Arsenic (As)	35.98 [19.52–55.60]	3.40 [2.26–4.57]	** < 0.001**
Zinc (Zn)	827.08 [557.9–1064.5]	749.15 [687.85–868.1]	0.299
Copper (Cu)	53.94 [44.20–75.16]	75.40 [72.60–84.02]	** < 0.001**
Iron (Fe)	502.97 [407.10–583.95]	753.35 [678.62–835.05]	** < 0.001**
Manganese (Mn)	15.32 [11.82–25.12]	7.15 [5.52–8.37]	** < 0.001**
Chromium (Cr)	46.87 [39.53–58.96]	4.90 [3.12–7.92]	** < 0.001**
Calcium (Ca)	64.00 [50.99–79.94]	82.55 [74.52–92.70]	** < 0.001**

Results are presented as median [first quartile – third quartile].

*MT* methadone treatment.

Bolded numbers represent significant differences.

Another interesting question is whether the levels of heavy metals are drug-use combination dependent among OUD patients or not. This was investigated by comparing the metal levels between different types of drugs abused (purely opium, opium & cannabis, opium & tramadol, and opium & cannabis & tramadol) (Table 3). The results showed no significant difference between drug-abused groups on all metals (Kruskal–Wallis test, *p* > 0.05). In other words, since the levels of metals were comparable between different groups, we can conclude that the abusing of other form of drugs with opium did not alter the concentrations of metals significantly among OUD patients.

**Table 3:** The comparison of the heavy metal levels (µg L^−1^) between drug-used groups.

Element	Opium	Opium & cannabis	Opium & tramadol	Opium & cannabis & tramadol	P-value*
Calcium (Ca)	66.2 ± 19.6	70.6 ± 18.2	62.5 ± 17.3	64.0 ± 26.0	0.857
Chromium (Cr)	45.83 ± 14.9	51.1 ± 17.4	43.0 ± 8.1	49.3 ± 17.4	0.504
Manganese (Mn)	25.5 ± 18.0	20.6 ± 14.5	25.8 ± 23.8	20.2 ± 15.5	0.708
Iron (Fe)	525.1 ± 205.1	514.8 ± 156.0	496.3 ± 107.9	494.0 ± 144.3	0.839
Copper (Cu)	64.7 ± 22.7	52.9 ± 20.4	61.4 ± 18.2	48.8 ± 25.8	0.134
Zinc (Zn)	862.9 ± 299	714.6 ± 310.3	1049.4 ± 363.6	726.4 ± 314.6	0.065
Arsenic (As)	39.1 ± 19.0	41.1 ± 29.4	51.3 ± 20.1	37.6 ± 25.3	0.401
Selenium (Se)	52.7 ± 21.4	50.9 ± 25.8	50.6 ± 27.3	53.2 ± 27.2	0.995
Cadmium (Cd)	2.7 ± 1.5	2.8 ± 1.4	2.4 ± 1.7	2.8 ± 1.0	0.808
Lead (Pb)	38.6 ± 15.4	38.8 ± 18.1	40.7 ± 14.2	30.8 ± 16.5	0.191

*P-value was obtained using Kruskal–Wallis test.

Table 4 shows the result of the robust regression analysis for each metal as a dependent and age, group indicator (OUD/non-OUD), sex, cigarette smoking habit (yes/no), education (less than highschool, highschool, university), and occupation (yes/no) of individuals as independents variables. Table 4 reports the coefficients of this model with the significant coefficients as highlighted in boldface. After adjusting other variables, with the exception of As, the effect of age was no longer significant on other metal/elements. However, the differences between the concentrations of all metals (except Zn) between two groups were still remained significant after adjusting for all variables (*p* < 0.05).

**Table 4:** The coefficient estimates of the effect of covariates under robust regression analysis.

Covariate	Trace elements									
	Ca	Cr	Mn	Fe	Cu	Zn	As	Se	Cd	Pb
Age	−0.004	0.001	−0.003	−0.001	−0.001	0.004	**0.015**	−0.003	−0.008	−0.002
Group (control)	**0.206**	**−2.23**	**−0.912**	**0.422**	**0.331**	−0.004	**−2.273**	**0.398**	**−1.25**	**−1.92**
Sex (male)	−0.018	0.005	−0.056	−0.001	−0.054	−0.109	−0.224	−0.048	−0.032	0.025
Smoking (yes)	−0.814	−0.099	−0.009	−0.040	0.016	0.099	−0.068	0.028	−0.155	0.032
Education										
High school	0.048	0.137	0.126	0.032	0.029	−0.001	0.095	−0.004	−0.048	0.025
Academic	0.108	0.056	−0.200	−0.017	−0.019	0.039	0.182	0.081	−0.054	−0.073
Occupation (yes)	0.034	−0.119	0.132	0.048	0.041	−0.004	0.098	−0.046	0.013	0.098

Numbers in boldface were significant at 0.05 level.

### Correlation analysis

Table 5 and 6 show the correlation matrix of the trace element concentrations for the control and methadone treated group respectively. Different patterns of correlations between trace elements can be seen between two groups. While there was no significant correlation between elements in the control group (except for a weak Spearman correlation between Ca–As, ρ = 0.283), Some strong and positive associations can be seen between pairwise concentration levels in the MT group. The correlation of Pb with other metals is notable. Fe–Mn (ρ = 0.688), Zn-Cu (ρ = 0.588), and Zn-As (ρ = 0.418) are also noticeable.

**Table 5:** Correlation matrix of the heavy meal concentrations in control group.

	Ca	Cr	Mn	Fe	Cu	Zn	As	Se	Cd
Cr	0.137								
Mn	0.031	0.025							
Fe	−0.181	0.181	−0.145						
Cu	−0.009	−0.074	0.062	−0.223					
Zn	0.046	−0.144	−0.137	−0.007	−0.040				
As	0.283*	0.052	−0.069	−0.051	0.144	0.156			
Se	0.080	−0.110	0.158	−0.093	0.0004	0.181	0.132		
Cd	0.061	0.133	0.039	−0.036	−0.183	−0.014	−0.137	0.109	
Pb	0.039	−0.220	0.014	−0.215	0.037	0.131	−0.003	−0.027	0.016

Bolded numbers represent significant correlations (*p < 0.05).

**Table 6:** Correlation matrix of the heavy meal concentrations in methadone treated group.

	Ca	Cr	Mn	Fe	Cu	Zn	As	Se	Cd
Cr	0.144								
Mn	0.047	−0.001							
Fe	0.164	0.355**	0.688***						
Cu	0.184	0.299*	0.192	0.439***					
Zn	−0.047	0.038	0.131	0.219	0.588***				
As	0.189	0.024	0.181	0.228	0.321**	0.417***			
Se	0.193	0.114	−0.343**	−0.274*	−0.084	−0.281*	−0.133		
Cd	0.117	−0.056	0.076	0.004	−0.210	−0.221	−0.036	0.038	
Pb	0.255*	0.307*	0.229	0.345**	0.411***	0.244*	0.355**	−0.126	−0.129

Bolded numbers represent significant correlations (*p < 0.05, **p < 0.01, ***p < 0.001).

## Discussion

In this study comparing individuals on MT and persons without OUD, our data suggest that urinary concentrations of Se, Cu, and Fe levels were significantly lower in the MT group compared to the healthy subjects. But the concentrations of Mn and Ca in the MT group were higher than in the healthy group.

Various factors may be responsible for changing the levels of essential trace elements. Many studies of this population have evidenced that the nutritional status of the persons with OUD before and during the MT program can be changed. They may have deficient feeding and nutrient intake^29–33^. For example, Szpanowska-Wohn et al. demonstrated that after 2 months of methadone treatment, the daily intake of some minerals, vitamins, fats, dietary fiber, saturated fatty acids, and essential fatty acids was reduced^30,32^. Also, the side effects of methadone and opioids, including anorexia, constipation, abdominal pain, and vomiting can affect the function of the gastrointestinal tract and induce various mineral deficiencies in the body as well as malnutrition^7,34^. Also, the variations in essential trace element concentrations may be due to smoking habits, inflammation, and peripheral responses of the body to increased oxidative stress due to OUD and/or withdrawal syndrome of the cessation process^7,35^.

Some studies investigated the essential trace elements levels in persons with OUD, with partly conflicting results^2,9,36,37^. The data in the context of methadone treatment is limited and contradictory. In a study, random urinary samples from people treated with methadone and healthy subjects were assessed for Zn, Cu, and Ca. The excretion of Cu in patients on methadone treatment decreased, while that of Zn was excessive^38^. In another investigation, after one year of MT in persons with heroin use, the blood contents of Zn and Ca increased, whereas the Cu and Mg showed no significant changes^22^. Some others reported that the serum concentration of Cu decreased after methadone treatment^2,10^. The levels of selenium in urine samples of about 40% of subjects on methadone treatment were a little lower than levels get by the healthy subjects^24^.

Díaz-Flores et al. (2004) determined trace element levels in persons with heroin use who entered and did not enter the MT program. No significant differences were reported in the most of serum trace element levels due to the methadone detoxification. Just the serum Zn levels of patients who participated in MT were higher than those observed in patients not entered the MT program, although the difference was not significant. This treatment increased the Mg levels compared to healthy people. The serum levels of Zn and Fe tend to normalize with the increased years of MT, and the Se and Cu concentrations were not improved^39^. The major findings of Cemek’s study were that the trace element concentrations were affected by the variable and fixed doses of heroin administrations or opioid withdrawal syndrome^35^. In heroin withdrawal syndrome animals, Fe, Mn, Mg, and Ti concentrations decreased but Cu, Ca, and Al, concentrations increased^35^. Another study suggested a possible imbalance in micronutrients levels (significantly higher levels of Cu and Mg and lower levels of iron in serum), antioxidants, and immunoglobulin in persons with OUD, which tend to be returned to control values after methadone detoxification^2^. The different results in previous studies may be attributed to many factors such as socio-economic status, food preferences, the health status of participants, type and dosage of drug used, duration of methadone treatment, and sample size of different studies.

On the other hand, the effects of supplementation with micro elements on substance use and OUD recovery have been noted previously. The research data showed that minerals and vitamins supplementation in the detoxification period may have a positive effect, not only on malnutrition management but also as a relapse preventive measure and normalizer of physiological and biochemical status^7,21,40^. For example, a study reported that using magnesium in combination with methadone may help reduce relapse of opioid use disorder. Their results showed that after 12 weeks, the magnesium group had fewer relapses than the placebo group^41^. Also, zinc administration in patients with opioid use diorderhas been shown to reduce the dependence intensity, while zinc chelators can increase withdrawal symptoms in such patients^23,40^. Zn supplementation can reduce the likelihood of OUD relapse and ameliorate the mental health status of individuals undergoing MT^18^. Also, the analgesic effects have been reported for Zn administration^40^. It has been documented that OUDs affect dietary habits and, undermine appetite leading individuals dependent on drugs to desire foods that are low in nutrients and high in empty calories. Consequently, this can result in deficiencies of essential micronutrients^21^. Poor dietary habits and nutrition place people at increased risk of long-term health issues and immune deficiency. That is why drug users become susceptible to different infections^2^. Therefore, it is crucial to prioritize and monitor the nutrient intake and micronutrient levels of individuals undergoing MT. This focus on nutrition is essential for providing effective medical support, promoting recovery processes, and achieving positive outcomes in the recovery process.

Our results showed that the concentrations of Pb, Cd, As, and Cr in the MT group were higher than in the healthy group. It was predictable from the fact that patients undergoing MT had prior exposure to substances like opium, resulting in prolonged metal accumulation in their bodies. For example the extended half-life of lead, reported to be as long as 20 years, contributed to the expected outcomes.

Elevated levels of toxic elements due to OUDs have been documented by some investigators^37,42^ but the data concerning urinary concentrations of the studied toxic heavy metals during the MT program is limited in previous literature. A study conducted in Iran showed non-significant elevated levels of lead in the MT group compared to healthy controls. They also reported that the difference in blood lead levels between the patients under methadone therapy and persons with opium use was not significant. However, the lower lead levels in patients under MT than opium users, could represent a decreased risk of lead exposure after MT^25^.

The results of one study determined the amount of arsenic in the blood and urine samples of patients under the methadone treatment showed that about 50% of studied patients had an increase in the blood concentration of arsenic^24^. The accumulation of toxic metals in the urinary samples of participants on the methadone treatment may be correlated to previous history of drug use, high prevalence of cigarette smoking in SUD patients, and additional taking of toxicants of unknown origin.

Rahimi et al. (2020) investigated the lead content in methadone solution, opium tincture manufactured by pharmaceutical companies, and opium samples seized by the police, which are used for medicinal purposes. They found that lead was present in these samples but it was not at toxic levels^12^. It should be considered that this lead concentration can be dangerous in persons with OUD, who underwent MT due to daily intake of methadone oral solution, and opium tincture. Accumulation of toxic metals in the biological samples of patients with opioid use disorder undergoing MT may be due to metal adulterations of drugs and additional taking of toxicants from unknown sources.

The elevated levels of any of the above-mentioned toxic elements have a detrimental impact on the proper functioning of the human body. Heavy metals screening can be considered as one of the parts of multidimensional care for indivisuals on MT, aiming to assist them in recovery and prevent additional health problems. It is of great importance to alert healthcare professionals about the toxicological aspects associated with this matter. Healthcare providers and public health authorities should consider implementing strategies to mitigate potential adverse health effects associated with imbalances in essential and toxic elements identified in this study.

### Limitations

To interpret the current study’s findings, some limitations should be considered. There was a limited sample size in each subgroup and a larger study is needed to obtain more accurate estimates and reliable findings. We did not control for all potential factors that may have influenced the results, such as nutritional status, dietary supplement use, the severity of drug use, alcohol and cigarette use, duration of methadone use, and behavioral health problems such as anxiety. There is a need for a better understanding of essential trace elements and toxic heavy metals levels in different biological samples of individuals receiving MT considering differences between men and women or other subgroups, and changes over time. Changes in the urinary levels of trace elements in the current study cannot be attributed to methadone use or the previous drug use of the participants or the effects of both. More studies are needed considering the duration of treatment with methadone.

## Conclusion

Our results showed that urinary concentrations of Se, Cu, and Fe levels were significantly lower in the MT group compared to the healthy subjects. But the concentrations of Pb, Cd, As, Mn, Cr, and Ca in the MT group were higher than in the healthy group. Therefore, there is a need for nutritional support and toxic element monitoring in MT patients which should be incorporated into harm reduction interventions. Larger studies are needed to follow up on this important finding. The findings have direct implications for harm reduction interventions, clinical practice, and public health interventions, as they provide valuable insights into the potential nutritional and toxicological consequences of OUD in patients who are under methadone treatment.

## Data Availability

The data used and analyzed for the current study are available from the corresponding author upon request.

## References

[1] Heath, M.M. *Nutrient and Food Component Supplementation for Substance Use Disorder Recovery: A Systematic Review* (2019).

[2] Mannan SJ, Azad MAK, Ullah M (2011). Investigation of serum trace element, malondialdehyde and immune status in drug abuser patients undergoing detoxification. Biol. Trace Elem. Res..

[3] Truong TT, Kosten TR (2022). Current status of vaccines for substance use disorders: A brief review of human studies. J. Neurol. Sci..

[4] Lappan SN, Brown AW, Hendricks PS (2020). Dropout rates of in-person psychosocial substance use disorder treatments: A systematic review and meta-analysis. Addiction.

[5] Danaei G, Farzadfar F, Kelishadi R (2019). Iran in transition. Lancet.

[6] Shahbazi F, Mirtorabi D, Ghadirzadeh MR (2020). Years of life lost (Yll) due to substance abuse in iran, in 2014–2017: Global burden of disease 2010 method. Iran. J. Public Health.

[7] Mahboub N, Rizk R, Karavetian M (2021). Nutritional status and eating habits of people who use drugs and/or are undergoing treatment for recovery: A narrative review. Nutr. Rev..

[8] Cheng F-L, Wang H, Wu J (2005). Determination and correlation analysis of trace elements in hair of dependence drug addicts. Guang pu xue yu Guang pu fen xi Guang pu.

[9] Hossain KJ, Kamal MM, Ahsan M (2007). Serum antioxidant micromineral (Cu, Zn, Fe) status of drug dependent subjects: Influence of illicit drugs and lifestyle. Subst Abuse Treat Prev Policy.

[10] Piekoszewski W, Pach J, Sadlik K (2000). Changes in serum copper level during detoxification of acutely poisoned drug addicts. Biol. Trace Elem. Res..

[11] Nozadi F, Azadi N, Mansouri B (2021). Association between trace element concentrations in cancerous and non-cancerous tissues with the risk of gastrointestinal cancers in Eastern Iran. Environ. Sci. Pollut. Res..

[12] Rahimi M, Eshraghi MA, Shadnia S (2020). Lead contamination in opium, opium tincture, and methadone oral solution, in Iran. Addict. Health.

[13] Rezaei M, Javadmoosavi SY, Mansouri B (2019). Thyroid dysfunction: How concentration of toxic and essential elements contribute to risk of hypothyroidism, hyperthyroidism, and thyroid cancer. Environ. Sci. Pollut. Res..

[14] Dehghanifiroozabadi M, Noferesti P, Amirabadizadeh A (2019). Blood lead levels and multiple sclerosis: A case–control study. Multiple Scler. Relat. Disord..

[15] Mansouri B, Majnoni F (2014). Comparison of the metal concentrations in organs of two bird species from western of Iran. Bull. Environ. Contamin. Toxicol..

[16] Sayadi MH, Pavlaki MD, Martins R (2021). Bioaccumulation and toxicokinetics of zinc oxide nanoparticles (ZnO NPs) co-exposed with graphene nanosheets (GNs) in the blackfish (*Capoeta*
*fusca*). Chemosphere.

[17] Mansouri B, Pourkhabbaz A, Ebrahimpour M (2013). Bioaccumulation and elimination rate of cobalt in *Capoeta fusca* under controlled conditions. Chem. Spec. Bioavail..

[18] Amini, Z. & Heidari Farsani, E. *Investigating the Effect of Zinc Supplementation on Probability of Relapse and Mental Health in Patients with Substance Abuse Disorder Undergoing Methadone Maintenance Treatment* (2022). 10.1186/s13011-023-00514-5 PMC9817328 36609286

[19] Sason A, Adelson M, Herzman-Harari S (2018). Knowledge about nutrition, eating habits and weight reduction intervention among methadone maintenance treatment patients. J. Subst. Abuse Treat..

[20] Friedmann PD, Schwartz RP (2012). Just call it “treatment”. Addict. Sci. Clin. Pract..

[21] Kolarzyk E, Szpanowska-Wohn A, Chrostek-Maj J (2010). Zinc, copper and magnesium intake in a daily diet in opiate-addicted persons before and after 5 years of methadone treatment. Probl. Higieny Epidemiol..

[22] Xing-wei Y, Yu-jin Y, Jun-ping G (2011). Trace element in blood among heroin addicts with methadone treatment. Chin. J. Public Health.

[23] Ciubotariu D, Ghiciuc CM, Lupusoru RV (2015). Experimental research showing the beneficial effect of oral zinc administration in opioid tolerance. Farmacia.

[24] Wietecha-Posłuszny R, Bednarek A, Kościelniak P (2005). Determination of selenium and arsenic in the blood and urine of patients taking part in a methadone programme. Probl. For. Sci. Z. Zagadnień Nauk Sądowych..

[25] Ghaemi K, Ghoreishi A, Rabiee N (2017). Blood lead levels in asymptomatic opium addict patients; A case control study. Emergency.

[26] Das S, Mitra K, Mandal M (2016). Sample size calculation: Basic principles. Indian J. Anaesth..

[27] Serdar CC, Cihan M, Yücel D (2021). Sample size, power and effect size revisited: Simplified and practical approaches in pre-clinical, clinical and laboratory studies. Biochem. Med..

[28] Kloke JD, McKean JW (2012). Rfit: Rank-based estimation for linear models. R J..

[29] Ii, S.S., Ryan, L. & Neale, J. Diet and nutrient intake of people receiving opioid agonist treatment (OAT): Implications for recovery. *Drugs Alcohol Today* **16**, 59 (2016).

[30] Szpanowska-Wohn A, Kolarzyk E, Pach D (2004). Nutritional status of opiate-dependent persons before and during methadone maintenance therapy. Przeglad Lekarski..

[31] Tomedi, L.E., Bodnar, L.M., Bogen, D. *et al*. Nutritional status of methadone‐treated pregnant women. *FASEB J*. **23**, 736.19–736.19 (2009).

[32] Szpanowska-Wohn A, Kolarzyk E, Pach D (2004). Intake of nutrients in daily nutritional ratios by opiate dependent persons during methadone maintenance therapy. Przeglad Lekarski.

[33] Wanjihia V, Muniu E, Mwangi M (2018). Comparison of nutritional status and food insecurity among people who inject illicit drugs, non drug users and those on methadone treatment in selected areas of Nairobi, Kenya. J. Alcohol Drug Depend..

[34] Leppert W. The impact of opioid analgesics on the gastrointestinal tract function and the current management possibilities [Polish version: Wpływ opioidowych środków przeciwbólowych na czynność układu pokarmowego oraz aktualne możliwości postępowania terapeutycznego p. 132]. *Contemp. Oncol./Współczesna Onkol*.**16**(2), 125–139 (2012). 10.5114/wo.2012.28792 PMC3687404 23788866

[35] Cemek M, Büyükokuroğlu ME, Hazman Ö (2011). Antioxidant enzyme and element status in heroin addiction or heroin withdrawal in rats: Effect of melatonin and vitamin E plus Se. Biol. Trace Elem. Res..

[36] Akbari A, Mosayebi G, Solhi H (2015). Serum zinc, copper, iron, and magnesium levels in Iranian drug users: A case control study. J. Addict. Med..

[37] Farnia V, Nakhaee S, Azadi N (2022). Comparison of urine trace element levels in tramadol addiction alone and its co-abuse with cigarette and opium in Western Iran. Environ. Sci. Pollut. Res..

[38] Iyengar V, Chou P, Costantino A (1994). Excessive urinary excretion of zinc in drug addicts: A preliminary study during methadone detoxification. J. Trace Elem. Electr. Health Dis..

[39] Díaz-Flores JF, Sañudo RI, Rodríguez EM (2004). Serum concentrations of macro and trace elements in heroin addicts of the Canary Islands. J. Trace Elem. Med. Biol..

[40] Ciubotariu D, Ghiciuc CM, Lupușoru CE (2015). Zinc involvement in opioid addiction and analgesia—Should zinc supplementation be recommended for opioid-treated persons?. Subst. Abuse Treat. Prev. Policy.

[41] Margolin A, Copenhaver M, Avants SK (2003). A preliminary, controlled investigation of magnesium l-aspartate hydrochloride for illicit cocaine and opiate use in methadone-maintained patients. J. Addict. Dis..

[42] Azadi N, Nakhaee S, Farnia V (2022). Multivariate statistical evaluation of heavy metals in the urine of opium individuals in comparison with healthy people in Western Iran. Environ. Sci. Pollut. Res..

